# Supplementary material for: Influence of Malignant Pleural Fluid from Lung Adenocarcinoma Patients on Neutrophil Response
Source: Cancers (Basel). 2022 May 20;14(10):2529. doi: 10.3390/cancers14102529 (PMC9139419; doi:10.3390/cancers14102529)
Supplement: Supplementary file 1 [file cancers-14-02529-s001.zip › cancers-1713599-supplementary.pdf]

cancers

Supplementary materials

Influence of Malignant Pleural Fluid from Lung Adenocarcinoma Patients on Neutrophil Response

Maria Mulet, Rubén Osuna-Gómez, Carlos Zamora, José M. Porcel, Juan C. Nieto, Lídia Perea, Virginia Pajares, Ana M. Muñoz-Fernandez, Nuria Calvo, Maria Alba Sorolla and Silvia Vidal

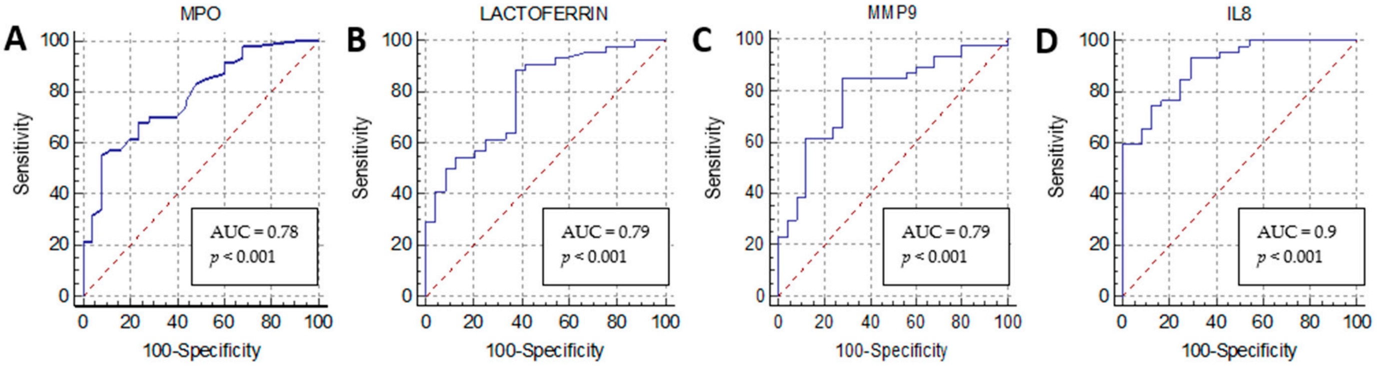

Supplementary Figure S1. Receiver operating characteristic (ROC) curves evaluating the diagnostic value of neutrophil-related factors in differentiating malignant from non-malignant pleural fluids. ROC curves were performed for **(A)** MPO, **(B)** lactoferrin, **(C)** MMP-9 and **(D)** IL-8 and their respective AUC and p-values are presented in the figure. AUC, Area under the curve.
